# Structure and activity of ChiX: a peptidoglycan hydrolase required for chitinase secretion by *Serratia marcescens*

**DOI:** 10.1042/BCJ20170633

**Published:** 2018-01-23

**Authors:** Richard A. Owen, Paul K. Fyfe, Adam Lodge, Jacob Biboy, Waldemar Vollmer, William N. Hunter, Frank Sargent

**Affiliations:** 1 School of Life Sciences, University of Dundee, Dundee DD1 5EH, U.K.; 2 Centre for Bacterial Cell Biology, Institute for Cell and Molecular Biosciences, Newcastle University, Newcastle upon Tyne NE2 4HH, U.K.

**Keywords:** anomalous dispersion, bacterial chitinase secretion, crystal structure, mutagenesis, peptidoglycan hydrolase, zinc enzyme

## Abstract

The Gram-negative bacterium *Serratia marcescens* secretes many proteins that are involved in extracellular chitin degradation. This so-called chitinolytic machinery includes three types of chitinase enzymes and a lytic polysaccharide monooxygenase. An operon has been identified in *S. marcescens*, *chiWXYZ*, that is thought to be involved in the secretion of the chitinolytic machinery. Genetic evidence points to the ChiX protein being a key player in the secretion mechanism, since deletion of the *chiX* gene in *S. marcescens* led to a mutant strain blocked for secretion of all members of the chitinolytic machinery. In this work, a detailed structural and biochemical characterisation of ChiX is presented. The high-resolution crystal structure of ChiX reveals the protein to be a member of the LAS family of peptidases. ChiX is shown to be a zinc-containing metalloenzyme, and *in vitro* assays demonstrate that ChiX is an l-Ala d-Glu endopeptidase that cleaves the cross-links in bacterial peptidoglycan. This catalytic activity is shown to be intimately linked with the secretion of the chitinolytic machinery, since substitution of the ChiX Asp-120 residue results in a variant protein that is both unable to digest peptidoglycan and cannot rescue the phenoytype of a *chiX* mutant strain.

## Introduction

*Serratia marcescens* is a Gram-negative opportunistic bacterial pathogen of the Enterobacteriaceae and is estimated to be responsible for ∼1.4% of nosocomial infections [1,2]. The pathogen relies heavily on the secretion of proteinaceous effector molecules including phospholipases, proteases, lipases and DNases, to compete and survive in the environment [3]. *S. marcescens* also secretes an array of chitinolytic proteins, together known as the ‘chitinolytic machinery’, including three chitinases (ChiA, ChiB and ChiC) [4–7], and a lytic polysaccharide monooxygenase (Cbp21) [8,9], which allows the utilisation of chitin as a carbon and nitrogen source and may also be important in the infection process for both chitinous and non-chitinous hosts [10].

The proposed mechanism of secretion of the extracellular chitinolytic machinery is an intriguing one. The ChiA and Cbp21 proteins are synthesised as precursors with N-terminal Sec signal peptides for export to the periplasm. However, ChiB and ChiC have no obvious canonical signal peptides to give clues to their initial targeting route. Using the *S. marcescens* DB10/DB11 strain as a model [1], genetic experiments isolated many mutant strains that are compromised in chitinase secretion [11]. In particular, a four-gene operon, subsequently named *chiWXYZ*, was identified that encoded proteins related to a bacteriophage lysis system [11]. The *chiW* gene encodes a predicted holin-like protein and the *chiX* gene encodes a predicted endolysin. Inactivation of either *chiW* or *chiX* impairs secretion of the entire chitinolytic machinery with the proteins accumulating in the periplasm unable to cross the outer membrane [11]. Moreover, the functions of ChiW and ChiX appear to be intimately linked. ChiW is predicted to be an inner membrane protein with three transmembrane domains, whereas ChiX is predicted to function in the periplasm, but does not itself contain an obvious signal peptide. Interestingly, the chitinase-secretion-defective phenotype of a *chiW* mutant can be rescued if the signal peptide from another protein is attached to ChiX [11]. This suggests that an important role of ChiW is in targeting ChiX to the periplasm of *S. marcescens* and that the localisation and biochemical function of ChiX is central to the secretion process.

This work presents a detailed structural and biochemical characterisation of ChiX. The high-resolution crystal structure of *S. marcescens* ChiX reveals that the protein belongs to the lysostaphin-type, d-Ala d-Ala metallopeptidases and sonic hedgehog ‘LAS’ family of Zn^2+^-dependent peptidases [12]. *In vitro* assays then establish ChiX as an l-Ala d-Glu endopeptidase that cleaves the cross-links in bacterial peptidoglycan, and site-directed mutagenesis is used to demonstrate that the chitinase secretion controlled by ChiX is directly attributable to this catalytic activity.

## Experimental procedures

### DNA manipulation

An overexpression system was developed to supply recombinant ChiX and the variant ChiX D120A protein. The *chiX* gene was amplified by PCR from *S. marcescens* DB10 genomic DNA to produce a fragment with engineered BamHI/NotI restriction sites, which were subsequently used to clone *chiX* into a pGEX-6P-1 vector (GE Healthcare) that would encode a fusion to glutathione *S*-transferase (GST). For expression of the native gene, a pBAD18 [13] construct was produced, with the *chiX* gene amplified by PCR to produce a fragment with engineered XbaI/SphI restriction sites before cloning into a pBAD18 vector. The D120A substitution was introduced into the pBAD18 *chiX* and pGEX6p1 *chiX* plasmids using the QuikChange Site-Directed Mutagenesis Protocol (Agilent).

### Recombinant protein production

The pGEX-6P-1 constructs allowed for IPTG-inducible production of ChiX and variants as N-terminal GST fusion proteins with PreScission protease sites located between the GST and target proteins. *Escherichia coli* BL21 (GOLD) harbouring the plasmids was grown at 37°C in LB medium to an OD_600_ of 0.7. Protein production was induced by the addition of IPTG to a final concentration of 2 mM, the temperature reduced to 21°C and growth allowed to continue for a further 17 h. Cells were harvested by centrifugation at 4500×***g*** for 40 min at 4°C and the cell pellet was suspended in 10 ml of TBS (Tris–HCl-buffered saline, pH 7.6) buffer supplemented with DNAse (Sigma–Aldrich). Cells were lysed by passage through a homogeniser (EmulsiFlex-C3) three times. The cell lysate was then clarified by centrifugation (40 000×***g*** at 4°C) for 45 min. The supernatant was passed through a 0.45 µM filter to remove remaining particulates before incubation with 1 ml of TBS equilibrated glutathione 48 sepharose 4B beads (GE Healthcare) with shaking for 2 h at 4°C. The lysate bead mixture was loaded into an empty gravity flow column and washed with 2 × 10 column volumes cold TBS buffer. The fusion polypeptide was then cleaved in 5 ml of TBS supplemented with 1 : 50 molar ratio of PreScission : protein at 4°C with gentle shaking for 16 h. This mixture was then loaded into a gravity flow column and the flow-through, containing cleaved target protein, collected and analysed by SDS–PAGE. Proteins were then concentrated to 12 mg ml^−1^ by centrifugation using a Vivaspin 10 000 MWCO concentrator (Sartorius) and further purified by size-exclusion chromatography using a HiLoad 16/60 Superdex 75 column (GE Healthcare) equilibrated with TBS buffer. Well-defined peaks obtained during size-exclusion chromatography corresponded to the ChiX (Supplementary Figure S1) and ChiX D120A monomers (15 kDa). SDS–PAGE analysis demonstrated the high level of purity for the samples (Supplementary Figure S1) and electrospray mass spectrometry confirmed the D120A substitution (Supplementary Figure S2). Protein concentrations were determined by absorbance at 280 nm using the predicted molar extinction coefficient of 27 960 M^−1^ cm^−1^ (PROTPARAM, [14]).

### ChiX crystallisation

Initial crystallisation screens were carried out at room temperature using the sitting-drop vapour-diffusion method in 96-well plates. This was achieved using a Phoenix liquid-handling system (Rigaku, Art Robbins Instruments) and commercially available screens. Crystallisation was observed in several conditions, but attempts to optimise the diffraction using vapour-diffusion methods failed. Crystals were tested through the screening and optimisation process using the in-house X-ray generator (Rigaku M007HF with a Saturn 944HG+ CCD detector). Eventually, streak seeding was utilised to improve crystal quality. The optimised condition was formed by adding 1 µl of ChiX at 3.4 mg ml^−1^ in 0.02 M Tris–HCl (pH 7.6), 0.15 M NaCl, to 1 µl of the reservoir, which consisted of 0.1 M Tris–HCl (pH 7.5), 0.14 M trimethylamine *N*-oxide and 16% (w/v) polyethylene glycol 2000 monomethyl ether. Crystals consisted of rectangular and triangular prisms with maximum dimensions of ∼60 µm and grew over 2 days. A suitable cryoprotectant was identified (40% polyethylene glycol 400).

### X-ray data collection and structure determination

Two isomorphous crystals were used for data collection, termed ChiX-1 and ChiX-2, at the European Synchrotron Radiation Facility (ESRF), Grenoble, France (Table 1). X-ray absorption near edge structure (XANES) spectroscopy with an excitation range of 9260–9740 eV was used to confirm the presence of Zn^2+^ in ChiX-1 (Supplementary Figure S3). The peak wavelength of this scan was determined as 9669 eV (1.28 Å). As no Zn^2+^ was added during the purification and crystallisation of ChiX, it most probably originated from the growth medium. Data were measured at the peak wavelength, ChiX-1, to optimise the *f*’′ signal for structure solution. A second dataset was measured at a shorter wavelength of 0.98 Å, ChiX-2, to produce high-resolution data with improved statistics for refinement.

**Table 1:** Crystallographic statistics

	Dataset 1		Dataset 2	
Diffraction data				
X-ray source	ESRF	ID23-1	ESRF	ID23-1
Detector	Pilatus	6M-F	Pilatus	6M-F
Wavelength (Å)	1.282		0.977	
Detector distance (mm)	147.541		262.164	
Exposure time (s)	0.037		0.037	
Δ*Φ* (°)	0.1		0.1	
Total *Φ* (°)	300		360	
Temperature (°C)	−173		−173	
Space group	*P*2_1_		*P*2_1_	
Cell dimensions *a*, *b*, *c* (Å), *β* (°)	40.56, 55.50, 51.60, 90.16		40.63, 55.53, 51.78, 90.00*	
No. of observations	263 714		322 385	
No. of unique observations	49 738		50 019	
Resolution overall (Å)	55.50–1.34		55.53–1.34	
Resolution highest bin (Å)	1.36–1.34		1.36–1.34	
Multiplicity	5.3 (3.8)		6.9 (6.1)	
Anomalous multiplicity	2.6 (2.0)			
Completeness	96.8 (83.0)		90.5 (50.4)	
Anomalous completeness	94.4 (77.3)			
Wilson *B*-factor	62.2		55.1	
〈*I/σI*〉	10.5 (1.3)		6.7 (2.3)	
*R*_merge_	0.082 (0.92)		0.084 (0.52)	
Mn(I) *CC*(1/2)	0.997 (0.36)		0.998 (0.83)	
Refinement				
*R*_work_/*R*_free_ start	0.2869/0.2755		0.1253/0.1394	
*R*_work_/*R*_free_ end	0.1334/0.1638		0.089/0.12	
Protein residues	1–266		1–266	
Zn^2+^			2	
Waters			323	
RMSD from ideal geometry				
Bond lengths (Å)			0.013	
Bond angles (°)			1.66	
Thermal parameter (*B*) values (Å^2^)				
Mean *B* atoms/protein/water/Zn^2+^			12.7/11.1/21.3/8.0	
Ramachandran				
Favoured/allowed/outliers (%)			99.31/0.69/0	

*A rare example of a monoclinic system with *β* = 90.0°.

All data were indexed and integrated using XDS [15] and scaled using Aimless [16]. Estimates of the Matthews's coefficient (2 Å^3^/Da) and bulk solvent content (40%) suggested two molecules *per* asymmetric unit. Using the software pipeline CRANK2 [17], Zn^2+^ positions were identified, experimental phases calculated, followed by density modification and automatic structure building to produce the first model (figure-of-merit 0.63). The model, electron and difference density maps were inspected using Coot [18]. Limited refinement was carried out using REFMAC5 [19], interspersed between cycles of interactive model building against the difference and electron density maps. Once the model was almost complete, it was used for rigid body refinement followed by several cycles of manual model building using the ChiX-2 dataset. The maps were significantly improved, especially around the Zn^2+^ positions. Furthermore, other statistics such as 〈*I/σI*〉, *R*_merge_ and the correlation coefficient *CC*_1/2_ in the outer shell were superior.

The two ChiX molecules in the asymmetric unit were refined independently. A Hamilton *R* ratio test was carried out using the PDB_REDO server [20] and suggested that anisotropic refinement of thermal parameters should be carried out. Henceforth, discussion concerns the model obtained from the high-energy dataset unless stated otherwise. MolProbity [21] was used to validate model geometry. Analyses of surface areas and interactions were made using the PISA [22] web service, and secondary structure analysis was performed using DSSP [23]. Crystallographic statistics are presented in Table 1. The structure factors and co-ordinates for the ChiX crystal structures have been deposited in the PDB under accession codes 5OPZ and 5OQ1.

### Peptidoglycan hydrolysis assay

Purified sacculi from *E. coli* D456 lacking PBP4, PBP5 and PBP6 [24], containing monomeric and cross-linked dimeric and trimeric tetrapeptides, tripeptides and pentapeptides, were incubated with purified ChiX (or ChiX D120A) at a range of concentrations, alongside no enzyme control samples. Additional ZnCl_2_ or EDTA was included where indicated. Incubations were carried out at for 4 h at 37°C, before samples were inactivated by 10 min boiling. Soluble muropeptides were then obtained by digestion with cellosyl (overnight, 37°C), boiling for 10 min and centrifugation. Samples were reduced with sodium borohydride and analysed by high performance liquid chromatography (HPLC), as described in Chou et al. [25]. Some of the HPLC profiles were obtained with a shorter (70 min) gradient, and the other parameters were unchanged (chromatograms in Figure 4). Selected fractions were then analysed by ESI–MS/MS to determine their composition [26].

### Complementation of mutant phenotypes

An *S. marcescens* Δ*chiX* strain (JJH05x [11]), possessing an unmarked in-frame deletion for *chiX*, was transformed with either the pBAD18 *chiX* or pBAD18 *chiX D120A* plasmid. The strains were grown at 30°C for 16 h with 0.02% (w/v) l-arabinose before being separated into whole-cell and supernatant samples, as described in ref. [11]. These samples were then separated by SDS–PAGE and analysed by Western immunoblotting using antisera for ChiC [11] and a commercial antibody against maltose-binding protein (MBP) (New England Biolabs).

## Results

### The crystal structure of *S. marcescens* ChiX

The *S. marcescens* ChiX protein was overproduced as a GST fusion in *E. coli* before being isolated, separated from GST by proteolysis and then finally subjected to size-exclusion chromatography (Supplementary Figure S1). The purified protein was entered into crystallisation trials and numerous conditions were found to produce small, poorly ordered crystals that were not suitable for data collection. A major problem was the presence of a large mosaic spread coupled to the presence of multiple lattices. A microseeding approach produced a significant improvement and resulted in samples suitable for structure determination. Owing to the presence of Zn^2+^ in ChiX (Supplementary Figure S3), the single-wavelength anomalous dispersion method was used to solve the structure.

The asymmetric unit contains two ChiX molecules (labelled A and B), and the model for each consists of ChiX residues 2–134, with residue 1 (serine) originating from the genetic construct. Only the N-terminal linker region originating from the pGEX-6P1 vector was unresolved in the electron density maps. The refinement did not employ any non-crystallographic symmetry restraints, and the root-mean-squared deviation (RMSD) of 0.17 Å for a least-squares overlay of 133 Cα positions indicates that the molecules are highly similar. Only molecule A will be discussed here, unless stated otherwise.

**Figure 1.**
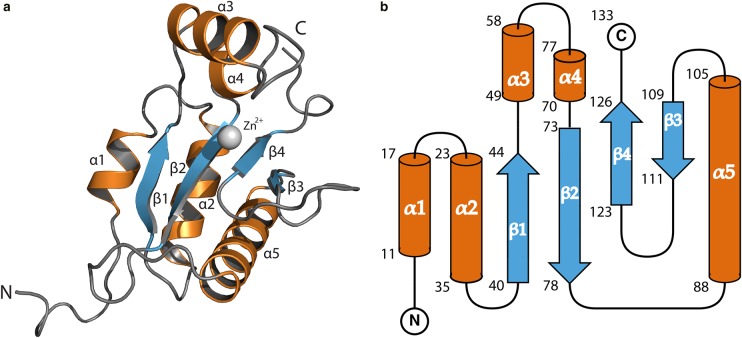
The structure of a ChiX monomer from *S. marcescens*. The ChiX molecule is an α/β protein. (**A**) Cartoon diagram depicting the ChiX molecule with α-helices, β-sheets and loops coloured orange, blue and grey, respectively. Bound Zn^2+^ is represented as a grey sphere. (**B**) A secondary structure schematic of ChiX, with equivalent colouring. The beginning and end residues of secondary structure elements are numbered.

ChiX presents an α/β-fold that consists of five α-helices, and four antiparallel β-strands are arranged as a single β-sheet in the order β1–β2–β4–β3 (Figure 1). Three helices (α1, α2 and α5) are placed on one side of the β-sheet. On the other side, and linking β1 and β2, α3 and a short α4 form a helical loop (Figure 1). This loop is orthogonal to the β-sheet, and together, they create an open and deep, mainly hydrophilic cleft. The Zn^2+^-binding site is at the base of this cleft and is primarily created by residues from one side of the β-sheet.

**Figure 2.**
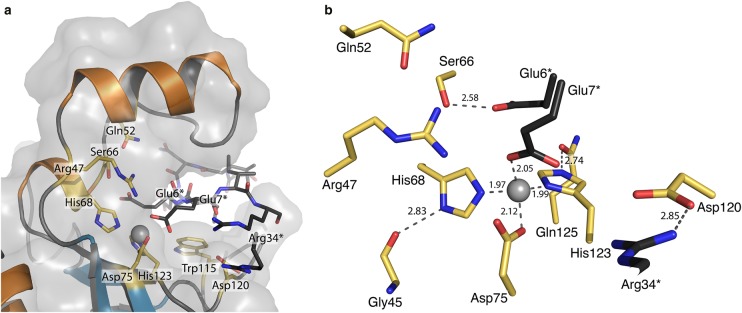
ChiX molecules contain a Zn^2+^ ion co-ordinated in the LAS configuration. ChiX contains Zn^2+^ tetrahedrally co-ordinated by His68, Asp75, His123 and (from the N-terminal region of the adjacent ChiX molecule) Glu7. (**A**) A cartoon, stick and transparent surface model highlighting residues that line the zinc-binding site cleft. Residues from the adjacent ChiX molecule in the asymmetric unit are coloured black. (**B**) Details of the Zn^2+^-binding site, showing co-ordination distances in Å, with the N-terminal residues of the adjacent molecule coloured black with asterisks.

The cation displays tetrahedral co-ordination geometry typical of many Zn^2+^-dependent enzymes involving two nitrogen and two oxygen atoms (e.g. [27]). Zn^2+^–N distances range from 2.05 to 2.12 Å, while the Zn^2+^–O distances range from 1.97 to 1.98 Å (Figure 2). Three of the co-ordinating residues are from one polypeptide and the fourth is provided by a different molecule. Two histidines, His68 contributed from α4 and His123 from β4, co-ordinate the metal through NE2 and ND1, respectively. The histidine side chain positions may be stabilised by donating hydrogen bonds from His68 ND1 and His123 NE2 to the carbonyl groups associated with the main chain of Gly45 and side chain of Gln125, respectively. The third co-ordinating group is Asp75 OE1 and the fourth is provided by Glu7 OE2 from the N-terminal tail of another ChiX molecule (molecule B) in the asymmetric unit (Figure 2). In the case of molecule B, co-ordination at the metal-binding site involves Glu7 contributed from a crystallographic symmetry partner A (Supplementary Figure S4). In either case, the Glu7 OE2 groups also accept hydrogen bonds donated by Arg47.

The interface between molecules A and B, that together represent the asymmetric unit, is dominated by hydrophilic interactions between the open cleft of molecule A and the N-terminal tail of molecule B (Supplementary Figure S4). In addition to the interactions involving Glu7, there are associations between Glu7–Ser66, Ile8–Ser60, Arg9 and Arg34 with Asp120 and numerous solvent-mediated linkages. There is, in addition, a pronounced hydrophobic stacking between Arg34 and Trp86 (Supplementary Figure S5).

### *S. marcescen*s ChiX is an l-alanyl d-glutamyl endopeptidase

**Figure 3.**
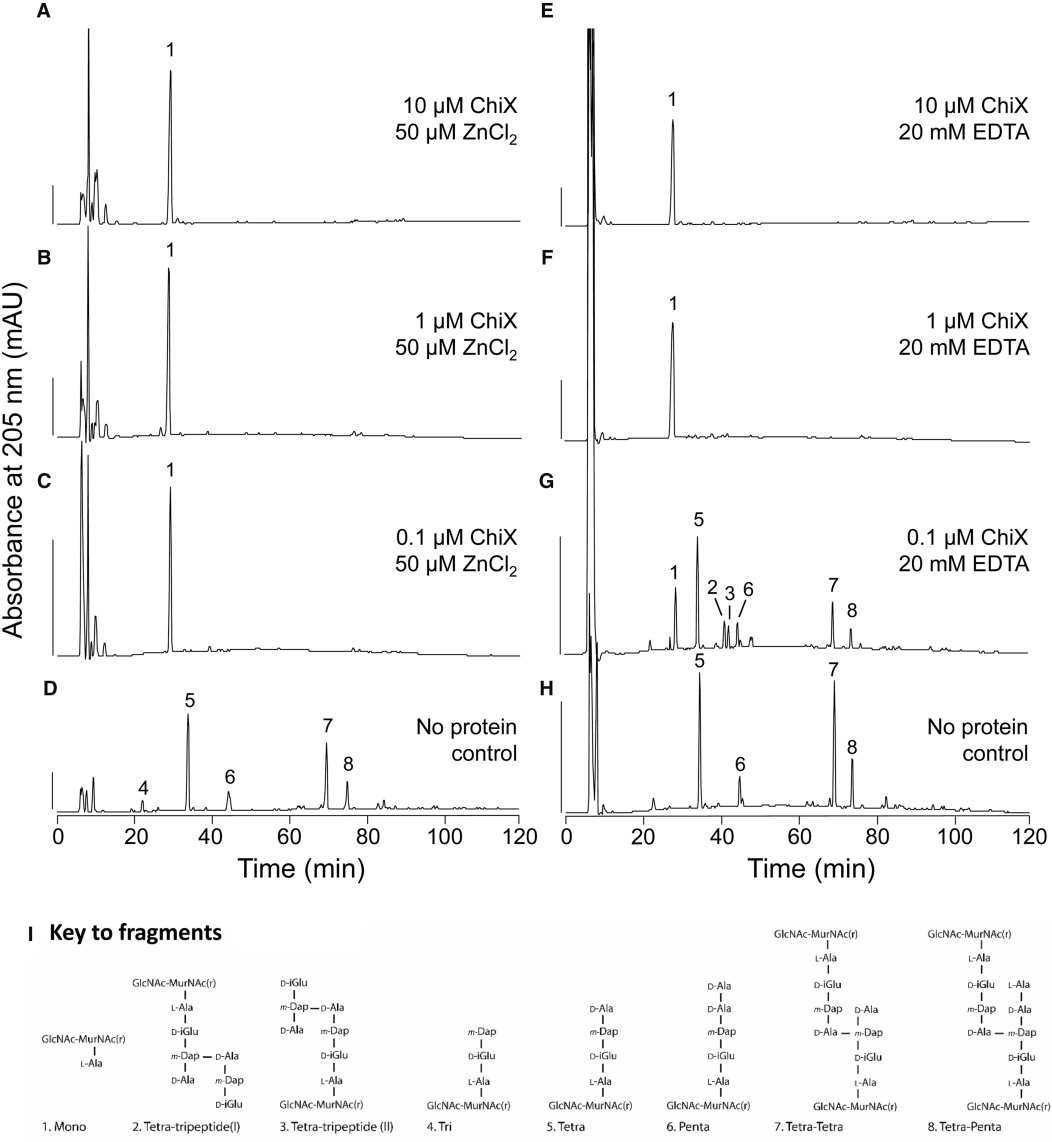
*S. marcescens* ChiX is an l-Ala d-Glu endopeptidase. The isolated ChiX protein was incubated with sacculi isolated from *E. coli* strain D456, followed by digestion with cellosyl and reduction with sodium borohydride. The resulting reduced muropeptides were separated by HPLC and selected fractions were analysed by ESI–MS/MS. (**A–D**) Peptidoglycan fragments were obtained with reducing amounts of ChiX in the presence of excess ZnCl_2_, as indicated. Scale bar, 200  mAU. (**E–H**) Peptidoglycan fragments obtained with decreasing amounts of ChiX incubated in the presence of excess EDTA, as indicated. (**I**) Proposed structures of peptidoglycan fragments corresponding to numbered fractions as they appear in the HPLC profiles.

Sequence comparisons suggested that ChiX might be a peptidoglycan hydrolase [11], and the purification protocol developed supplied material that allowed direct characterisation of the enzymatic activity. Peptidoglycan degradation assays were carried out to determine experimentally the precise cleavage position recognised by ChiX. Purified ChiX was incubated with purified sacculi from *E. coli* D456 that consisted of a mixture of tripeptides, tetrapeptides and pentapeptides in monomeric and cross-linked muropeptide subunits, with resultant HPLC chromatograms compared with a protein-free control sample (Figure 3). The results establish that native ChiX degrades the peptidoglycan fragments to a single mass species identifiable as GlcNAc–MurNAc–l-Ala (Figure 3A–C). This unequivocally classifies the enzyme as an l-Ala d-Glu endopeptidase (Figure 3).

The Zn^2+^-binding site is located at the base of a deep cleft (Figure 2) and, in the crystal structure, the cation is co-ordinated by a glutamate side chain contributed from a different ChiX protomer (Figure 2). Such an interaction possibly suggests that the zinc ion is important for recognition of substrate or catalysis by the l-Ala d-Glu endopeptidase. To test this directly, peptidoglycan cleavage experiments were carried out in the presence of high levels of EDTA (20 mM final concentration) in an attempt to inhibit activity by chelation of the Zn^2+^ (Figure 3E–H). This approach was only able to partially inhibit the enzyme when low amounts of protein were employed in the assay (Figure 3G), perhaps indicative of a combination of tight binding and restricted access to the cation by the chelator. The use of alternative chelators such as EGTA or BAPTA had no effect on ChiX activity (Supplementary Figure S6), although it should be noted that these have a lower affinity than EDTA for Zn^2+^ *in vitro*.

### Asp120 is critical for enzyme activity *in vitro* and *in vivo*

Secondary structure matching (SSM) [22] and Dali [28] were used to identify orthologous protein structures, with the most similar (RMSD 2.03 Å over 88%) being the C-terminal enzymatically active domain (EAD) of *Listeria* bacteriophage protein Ply500 (PDB code 2VO9; overall sequence identity with ChiX, 22% over 134 residues; *Z* score 7.5) [29]. Ply500 is predicted to be a Zn^2+^-dependent l-Ala d-Glu endopeptidase of the ‘LAS’ family of enzymes in which the cation is co-ordinated in a tetrahedral arrangement involving two histidines, an aspartate and a water molecule. As well as this metal-binding motif, the LAS family of enzymes also contain a catalytic side chain, normally an acid, which has a pivotal role in substrate hydrolysis [29]. For Ply500, the catalytic residue was suggested to be Asp130 [29], and superposition of the Ply500 metal-binding site with that of ChiX implicates that ChiX Asp120 could fulfil this role (Supplementary Figure S7).

**Figure 4.**
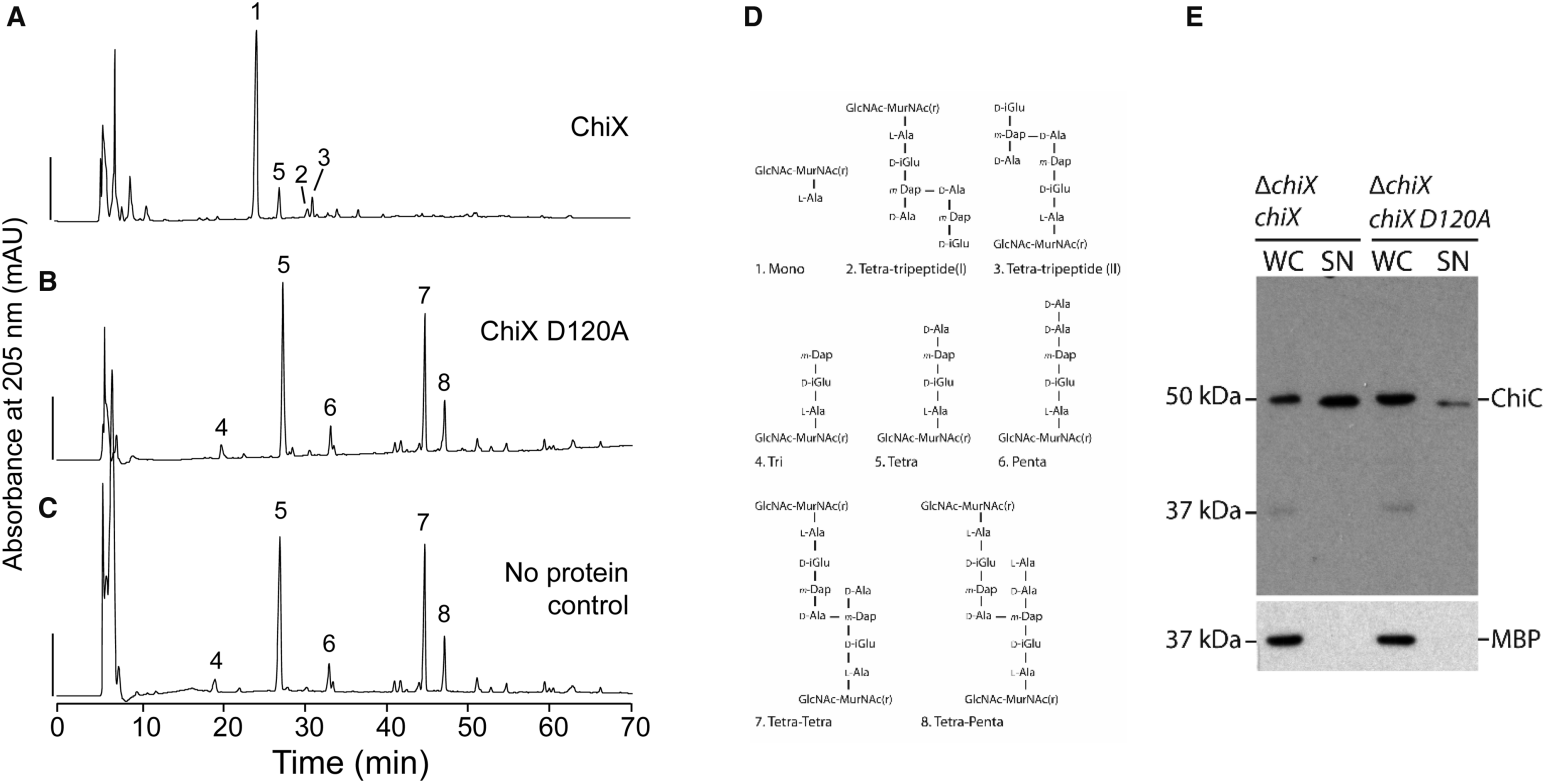
ChiX D120A is inactive and incapable of rescuing ChiC secretion in a Δ*chiX* mutant. The pGEX-6P-1 *chiX* vector was modified to code for a D120A variant of ChiX, which was then isolated from *E. coli* BL21(DE3) following the protocol devised for the native protein. (**A**) Native ChiX protein (0.01 µM) was incubated with sacculi isolated from *E. coli* strain D456 and resultant peptidoglycan fragments were digested with cellosyl, reduced and separated by HPLC. No additional zinc was added in this experiment. Scale bar, 200 mAU. (**B**) As-purified ChiX D120A (1 µM) was incubated with sacculi. (**C**) The HPLC profile of sacculi isolated from *E. coli* strain D456 without exposure to ChiX. (**D**) Proposed structures of peptidoglycan fragments corresponding to numbered fractions in the profile shown in (**A–C**). (**E**) *S. marcescens* strain JJH05x (Δ*chiX*) harbouring either pBAD18 *chiX* (‘Δ*chiX*, *chiX*’) or pBAD18 *chiX-D120A* (‘Δ*chiX*, *chiX D120A*’) was grown overnight in LB medium supplemented with 0.02% (w/v) l-arabinose. Cultures were then separated into whole cell (‘WC’) and culture supernatant (‘SN’) and analysed for ChiC secretion by SDS–PAGE and Western immunoblotting with anti-ChiC serum. A lysis control was also carried out by tracking the localisation of periplasmic MBP.

To explore this hypothesis further, experiments were carried out using a ChiX D120A variant (Figure 4). First, a variant enzyme was tested *in vitro* for peptidoglycan hydrolase activity (Figure 4). The ChiX D120A variant was overproduced and purified by a method identical with that used for native ChiX, and then assayed against a peptidoglycan substrate (Figure 4). The ChiX D120A variant was found to be unable to digest peptidoglycan and was therefore considered to be inactive (Figure 4). Next, a pBAD18 inducible plasmid encoding ChiX D120A was prepared and used to transform the *S. marcescens* JJH05x strain that carries a Δ*chiX* allele. After separating the culture supernatant and whole-cell components, samples were analysed by SDS–PAGE and Western immunoblotting using ChiC antisera (Figure 4). While a plasmid encoding native ChiX was able to rescue ChiC secretion to the Δ*chiX* mutant, the *chiX D120A* mutant was incapable of restoring ChiC secretion (Figure 4). Taken together, these data demonstrate that a D120A substitution in ChiX produces an inactive and non-functional protein product, thus establishing the Zn^2+^-binding site as the catalytic centre, a mechanistic contribution from Asp120 as an acid/base, and that the normal enzymatic activity of ChiX is required for chitinase secretion in *S. marcescens*.

## Discussion

Peptidoglycan-degrading enzymes are diverse in structure, mechanism and physiological role and can be found encoded within the genomes of bacteriophage, prokaryotes and eukaryotes [30]. Their biochemical activities allow classification as endopeptidases, amidases, glycosidases and lytic transglycosylases. Many of the enzymes that have been structurally characterised contain metals as cofactors. The bacteriophage T7 endolysin (T7 lysozyme), for example, requires Zn^2+^ for activity, but the metal is readily lost from the protein during purification [31]. This protein also has a pH-dependence to its structural integrity and activity, but it is not known if this is related to metal binding [32]. Some phage endolysins may retain the protein fold adopted by Zn^2+^-dependent enzymes, but lack both the metal and the classical catalytic residues identified in other systems, and therefore must cleave peptidoglycan by a novel mechanism [33]. Finally, some bacteriophage endolysins comprise multiple domains. For example, the LysK endolysin comprises three domains including cysteine–histidine-dependent amido-hydrolase/peptidase (CHAP) domain, an amidase domain and a cell wall-binding domain. The crystal structure of the CHAP domain revealed that both Zn^2+^ and Ca^2+^ were bound [34]. In this case, the catalytic cysteine and histidine side chains are very close to the Zn^2+^ and appear to act as ligands to the metal [34], placing the CHAP family enzymes in a different family to ChiX. A similar cysteine-dependent endolysin could be stripped of bound Zn^2+^ by dialysis in 5 mM EDTA, and this treatment was sufficient to completely inactivate the enzyme [35].

In this work, the *S. marcescens* ChiX protein has been revealed to be a Zn^2+^-dependent l-Ala d-Glu endopeptidase from the LAS family. The protein is encoded by a *bona fide* bacterial gene, rather than being located within a prophage integrated into the bacterial genome [11]. The enzymatic activity (Figure 4) and subcellular localisation [11] of this protein are critical for its physiological function. ChiX appears to be a monomer in solution, eluting from the size-exclusion chromatography column at a volume consistent with 15 kDa (Supplementary Figure S1). However, ChiX presents two molecules in the asymmetric unit of the crystal structure (Supplementary Figure S4). Analysis using the PISA server [22] calculated a surface area of ChiX as ∼8110 Å^2^, with an interface area of 950 Å^2^ (∼12%), and gave a Complex Formation Significance Score of 0.000, strongly suggesting that the dimeric assembly has no biological relevance and is an artefact of crystallisation. Nevertheless, the interface between the two molecules (Supplementary Figure S4) is formed through an interaction between the N-terminal loop region of one protein and the open cleft of another. The crystal packing shows that identical interactions are also present on the two ‘free sides’ of the asymmetric unit (Supplementary Figure S4). As each ChiX monomer forms two interfaces, extended chains of ChiX monomers are produced, with few additional interactions between alternative ChiX molecules present. It is likely that the interaction between the N-terminal extension of one protomer and the open cleft of the adjacent ChiX molecule is important for crystallisation. Moreover, given the interaction involves co-ordination of the Zn^2+^ ion by a glutamate side chain, it has the fortuitous benefit of informing on the mechanisms of substrate binding and hydrolysis.

### The ChiX active site and an inferred mechanism for hydrolysis

SSM [22] and Dali [28] were used to identify orthologous protein structures, and these including endopeptidases, carboxypeptidases and dipeptidases of differing specificity, and domains from Sonic Hedgehog proteins. The most similar structure identified was Ply500 (PDB: 2vo9) [29], for which a sequence/structure alignment was prepared (Supplementary Figure S7). Superimposing the two structures demonstrates conservation of the overall domain architecture (Supplementary Figure S7), with an RMSD value of 2.03 Å over 118 Cα atoms (*Z* score 7.5 and 22% sequence identity over the 134 resides of ChiX). Ply500 is classified as a Zn^2+^-dependent l-Ala–d-Glu–endopeptidase of the LAS family where the cation is co-ordinated by a tetrahedral arrangement of two histidines, an aspartate and a water molecule. ChiX clearly shares this arrangement around a centrally co-ordinated Zn^2+^ ion; however, in this case, the co-ordination provided by a water molecule is occupied by glutamate (Glu7) shared from the adjacent molecule within the asymmetric unit (Supplementary Figure S7) or with a symmetry-related mate.

While the conserved residues co-ordinate the metal ion, there are limited biochemical data on the significance of these residues in the mechanism of murein hydrolases beyond Zn^2+^ ligation. However, inspection of the VanX d-Ala d-Ala endopeptidase potentially gives some clues towards the mechanism [36]. For VanX (which has 16% overall sequence identity with ChiX; a *Z* score of 5.9 and RMSD 2.48), a protonated arginine (Arg71 in VanX and Arg47 in ChiX) was implicated in providing assistance for substrate binding, and the importance of Glu181 (Asp120 in ChiX) was highlighted as a proton abstractor from a Zn^2+^-polarised water molecule [36]. However, while data presented in this study clearly demonstrate that ChiX Asp120 is critical for enzymatic activity (Figure 4), the structural alignment with Ply500 indicates that ChiX Asp120 is pushed away from the metal ion relative to the equivalent Asp130 in Ply500 (Supplementary Figure S4). Indeed, while the closest Asp to Zn^2+^ distance is 4.0 Å in Ply500, it extends to 7.3 Å in ChiX (Supplementary Figure S4). This difference in conformation is potentially due to the salt bridge formed between Asp120 and Arg34 of the adjacent ChiX molecule in the asymmetric unit and further compounded by the presence of Glu7 also from the adjacent molecule, which forms the fourth co-ordination point with the Zn^2+^ and would prevent Asp120 moving closer due to electrostatic repulsion.

**Figure 5.**
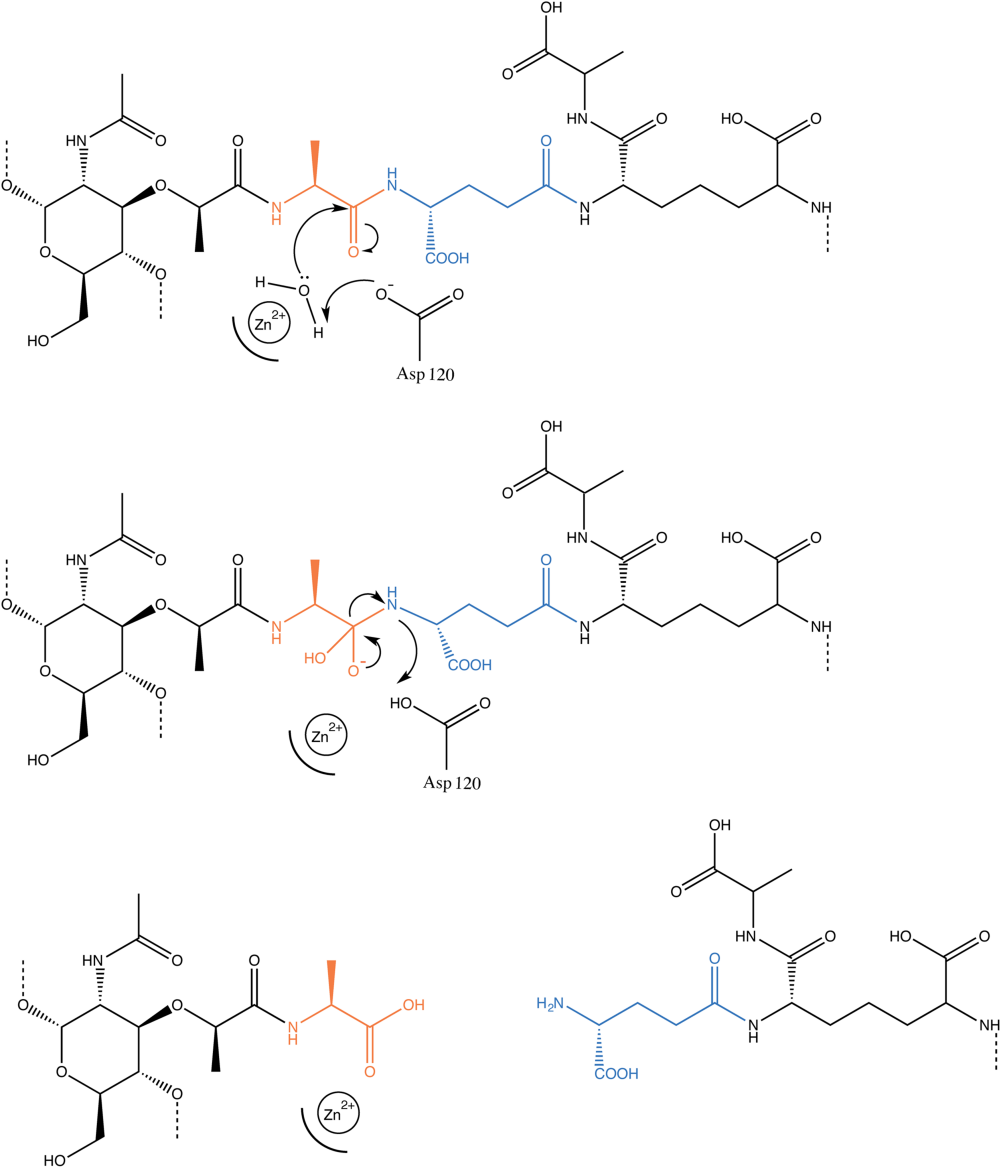
A possible mechanism for ChiX endopeptidase activity. The reaction mechanism is predicted to proceed through the nucleophilic attack on the carbonyl carbon by an activated water molecule. Water activation is likely to proceed through its proximity to the Zn^2+^, and possible deprotonation by Asp120. The resultant tetrahedral reaction intermediate is then resolved to complete the hydrolysis reaction.

With ChiX Asp120 established as an essential amino acid for enzymatic activity, a predicted ChiX reaction mechanism can be proposed (Figure 5). In the absence of the Glu7 artefact, present in the crystal form, it would be reasonable to expect that the fourth co-ordination site would occupied by water or an oxygen atom from the substrate or product as catalysis proceeds. The Zn^2+^ would act as a Lewis acid and hence polarise and activate the water molecule ready for proton abstraction by the nearby Asp120. An anionic hydroxide would be produced, which would carry out nucleophilic attack on the carbonyl carbon of the substrate peptide bond. An unstable five-membered reaction intermediate would then be formed and finally rapidly resolved to complete the hydrolysis reaction (Figure 5).

### Predicting the mode of substrate binding

**Figure 6.**
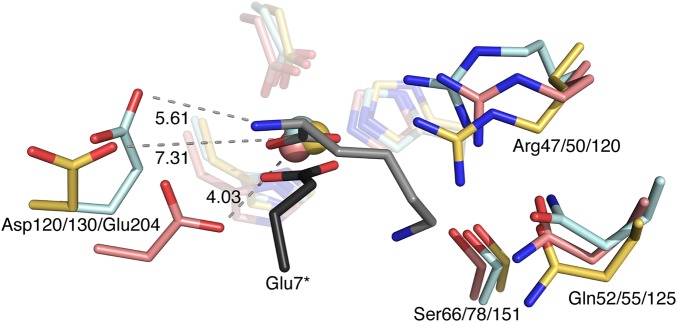
The substrate-binding site on LAS family enzymes. A comparison of the ChiX (yellow, with N-terminal Glu7 in black), Ply500 (pink) and LdcB (ligand bound, cyan and substrate grey) putative active sites. The numbering for Ply500 is from the EAD, which is at the C-terminus of Ply500, and reflects the numbering in Korndörfer et al. [29].

In terms of addressing substrate recognition and binding, the crystallographic pair formed by ChiX also gives some clues but, because of the occluded active site, also hampers further experimentation using traditional approaches such as soaking crystals with substrate analogues or fragments. However, there is a crystal structure of an LAS family protein that has a product mimic bound, the l-Lys d-Ala carboxypeptidase LdcB from *Streptococcus pneumoniae* [37]. When ChiX is compared with LdcB (overall sequence identity with ChiX 14%; *Z* score 7.5; RMSD 2.39) (Figure 6), the organisation of substrate around the Zn^2+^ is found to be similar, with one co-ordination point in both models occupied by a carboxylate, from Glu7 in ChiX and a remnant of the product mimic peptide bond in LdcB (Figure 6). Further to this, the LdcB Glu204 and ChiX Asp120 side chains appear to share similar conformations, both pointing away from their neighbouring Zn^2+^ ions. This is different from the conformation of the corresponding residue in Ply500, which has an empty exposed active site, and suggests that dynamic conformational changes performed by key residues in these proteins may contribute to activity.

In LdcB, the bound MurNAc sugar moiety is relatively distant from the metal-containing active site, and the orientation is potentially stabilised by hydrophobic interactions with Met202 [37]. In the case of ChiX, which cleaves the peptidoglycan at a different position to LdcB, it might be expected that the MurNAc group would be closer to the open cleft. Obvious candidates contributing to the formation of such a sugar-binding site could be the three tryptophan residues (Trp87, 89 and 116), which occupy the outer edge of the active site cleft (Supplementary Figure S5).

### The role of ChiX in protein secretion

Overall, this work adds important new structural and biochemical details on the unusual mechanism of chitinase secretion adopted by *S. marcescens*. Peptidoglycan hydrolases are enzymes that are commonly encoded by bacteriophage and prophage and designed to induce catastrophic cell lysis. Some microbes also display altruistic autolysis of subpopulations of cells, which is catalysed by endogenous lysins, and this is especially well understood in complex communities [38]. However, some enzymes have been described that are encoded by bacterial genomes and have roles in non-lytic protein secretion. Key examples have been described in *Salmonella typhi* [39], *Clostridium difficile* [40] and, in the case of ChiX, *S. marcescens* [11].

A mutant strain of *S. marcescens* devoid of ChiX was found to be impaired in the secretion of a select subset of ∼10 proteins, including all known chitinases and chitin-binding proteins [11]. In the Δ*chiX* mutant, the normally secreted enzymes were found to accumulate in the periplasm unable to cross the outer membrane [11]. Work described here has established that ChiX is a Zn^2+^-binding endopeptidase capable of severing the cross-links in peptidoglycan and thereby with the potential to alter the physical properties of the periplasm. How such activity facilitates the subsequent secretion of chitinases across the outer membrane remains largely unclear; however, it is possible that such an activity could allow components of a secretion system, some of which may remain to be discovered, to recognise each other and so assemble across the periplasm.

The tight binding of Zn^2+^ (the presence of 20 mM EDTA fails to completely inactivate the enzyme even in large molar excess, Figure 3E–H) suggests that the protein, which would acquire this cation in the cytoplasm, is folded, prior to targeting into the periplasm [41]. This is especially interesting as ChiX does not contain a canonical targeting peptide that we can recognise. Available evidence suggests that ChiX relies on its partner protein, the holin ChiW, to gain access to the periplasm and that this is the only role of the membrane-bound ChiW in chitinase secretion [11]. Future studies to delineate the complex relationship between holin proteins, such as ChiW, and their partner endolysins [42] will be necessary to advance the knowledge about chitin secretion by bacteria.

### Accession numbers

The co-ordinates and structure factor data for ChiX have been deposited in the PDB under the accession codes 5OPZ and 5OQ1.

## Abbreviations

CHAP: cysteine–histidine-dependent amido-hydrolase/peptidase
EAD: enzymatically active domain
GST: glutathione *S*-transferase
HPLC: high performance liquid chromatography
LAS: lysostaphin-type, d-Ala d-Ala metallopeptidases and sonic hedgehog
MBP: maltose-binding protein
SSM: secondary structure matching
