# Supplementary material for: Structure and activity of ChiX: a peptidoglycan hydrolase required for chitinase secretion by Serratia marcescens
Source: Biochem J. 2018 Jan 23;475(2):415–28. doi: 10.1042/BCJ20170633 (PMC5778951; doi:10.1042/BCJ20170633)
Supplement: Supplementary Figures [file BCJ-475-415-s1.pdf]

Structure and activity of ChiX, a peptidoglycan hydrolase required for chitinase secretion by *Serratia marcescens*.

Richard A. Owen, Paul K. Fyfe, Adam Lodge, Jacob Biboy, Waldemar Vollmer, William N. Hunter,

Frank Sargent

SUPPLEMENTARY INFORMATION

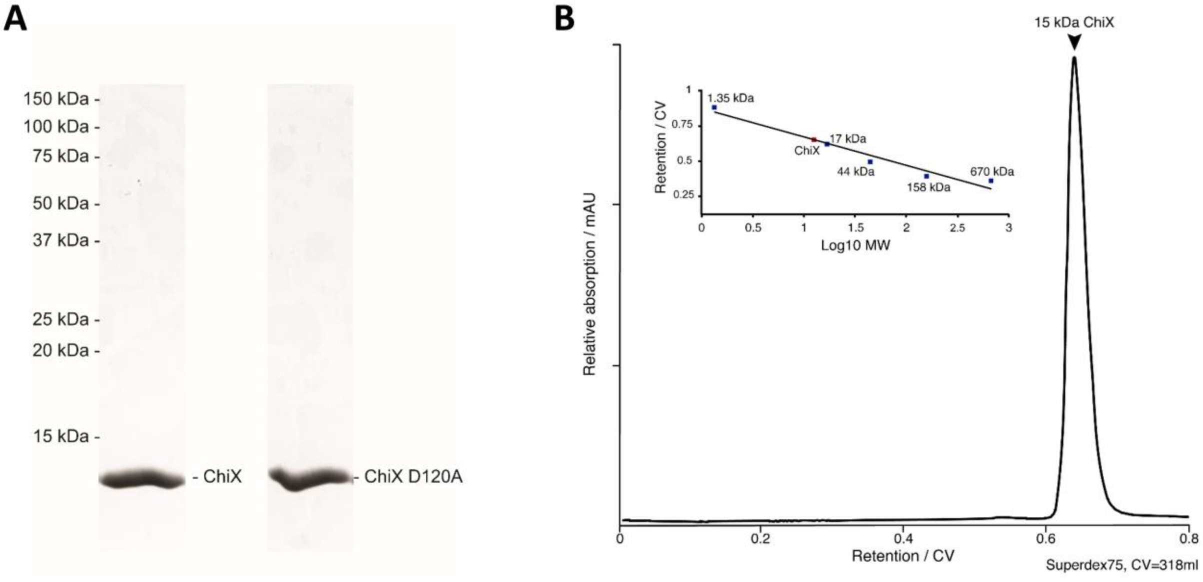

Supp. Figure S1: Biochemical analysis of purified ChiX and a D120A variant.
(A) Following production as GST-tagged fusion proteins the affinity tags were proteolytically removed and ChiX proteins recovered by revere affinity chromatography. The resultant ChiX and the ChiX D120A variant were assessed for potential contamination by denaturing SDS-Polyacrylamide Gel Electrophoresis. A 15% (w/v) acrylamide gel was prepared and, following separation of the proteins, was treated with InstantBlue™ Ultrafast Protein Stain (Sigma-Aldrich). **(B)** Size-exclusion chromatogram for native ChiX. Purified protein was loaded on to a column volume (CV) of 318 ml 26/60 Superdex 75 (prep grade; GE Healthcare) column equilibrated with 20 mM Tris.HCl, pH 7.6, 137 mM NaCl. A molecular mass of 15 kDa was calculated for ChiX using a previously determined calibration curve. The column was calibrated with molecular mass standards thyroglobulin, 670 kDa; γ-globulin, 158 kDa; serum albumin, 67 kDa; ovalbumin; 44 kDa, myoglobin, 17 kDa; and vitamin B_{12}, 1 kDa.

A. Native ChiX

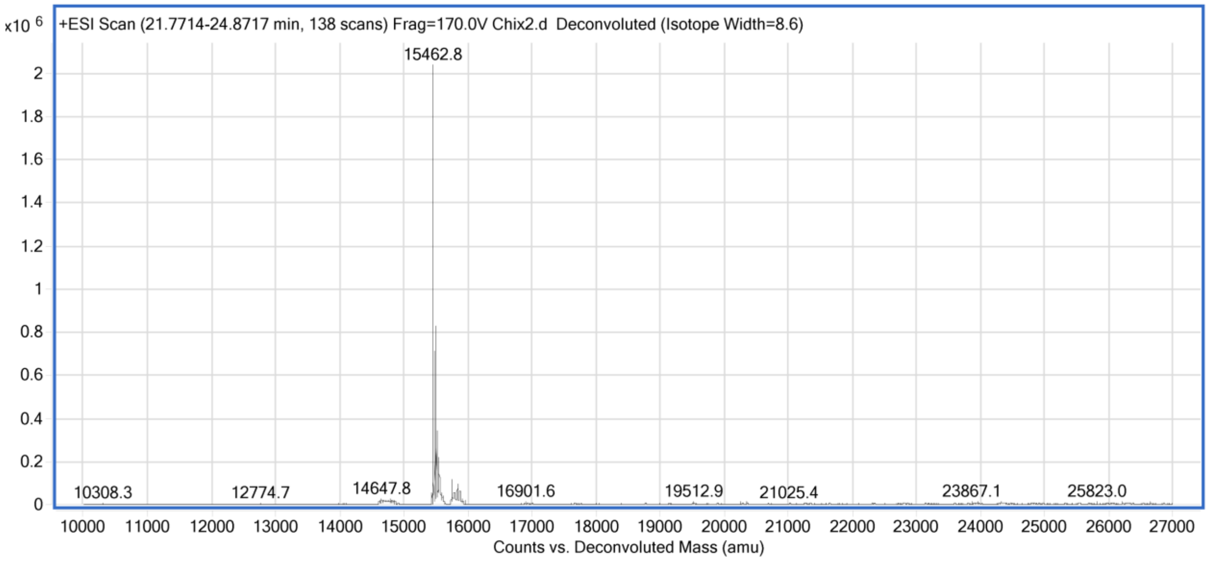

B. D120A ChiX

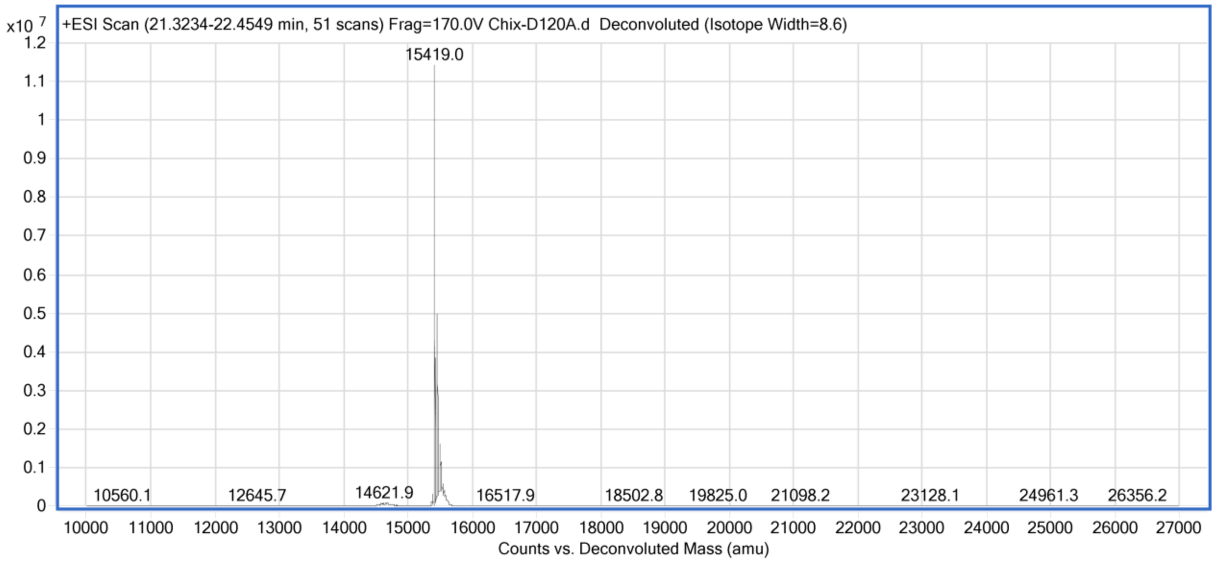

Supp. Figure S2: Analysis of recombinant ChiX and D120A ChiX by electrospray mass spectrometry. **(A)** Mass of native ChiX recorded at 15,462.8 Da, compared with a theoretical 15462.65 Da. **(B)** Mass of the D120A ChiX variant recorded at 15,419.0 Da, compared with a theoretical value of 15418.64 Da. Electrospray ionisation mass spectrometry was carried out on an Agilent 6520 Accurate-Mass QTof LC-MS system. Protein samples to be analysed were dialysed against pure water to remove any salts. Samples were introduced using a 1200 series nanoLC system (Agilent Technologies) and resolved on a C8 HPLC Chip (Zorbax 5µM 43mm × 75 µM, separation; 4mm, 40 nl enrichment) using a gradient of 5 – 70 % elution buffer (90% acetontrile in 0.08 % formic acid) over 10 minutes at a flow rate of 0.6 µl min^{-1}.

A

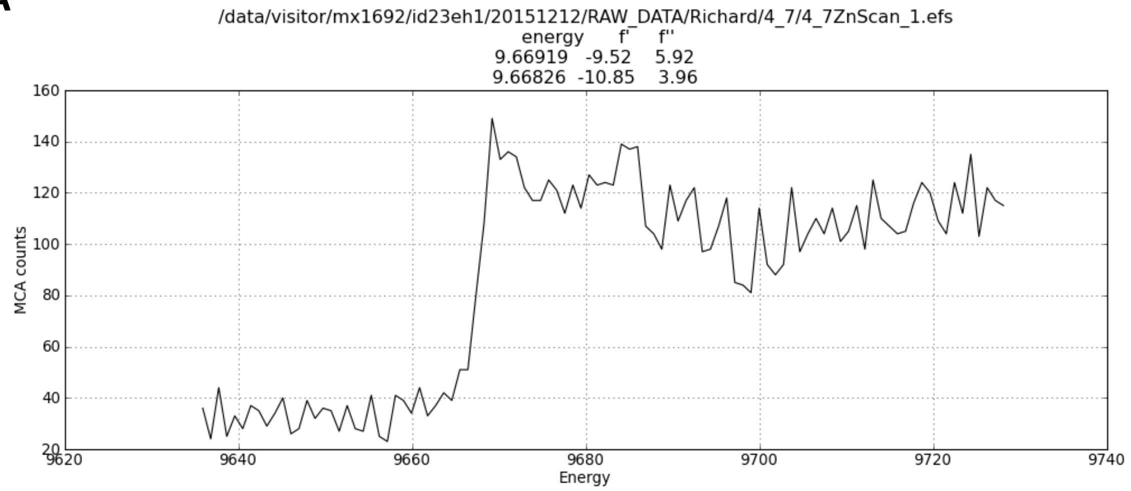

B

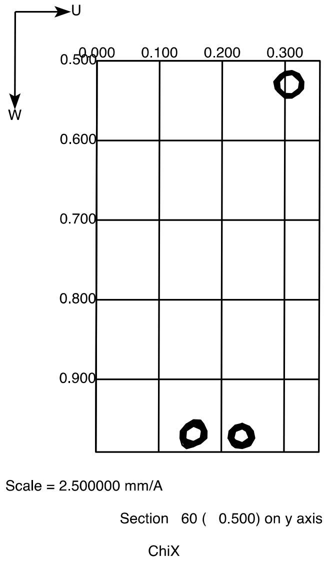

Supp. Figure S3: ChiX crystals contain Zn^{2+}

(A) XANES measurements were carried out at ESRF beam-line ID23-1 with a Rontec Silicon Drift Diode, and showed a fluorescence emission corresponding to the wavelength of Zn^{2+} excitation. **(B)** ChiX-Zn contains four Zn^{2+} atoms *per* unit cell. Harker section (y=1/2) suggests the presence of four ordered Zn^{2+} atoms in the unit cell. The sofware FFT (CCP4 suite) (Schoenmyer, Tor; Zhang, David Y. "FFT-based Algorithms for the String Matching with Mismatches Problem." J. Algorithms 57(2): 130-139 (2005)) was utilised to produce a Harker section of the anomalous Patterson map calculated with anomalous data collected from ChiX-1. Contour levels from 3.52 σ to 10 σ in intervals of 1.

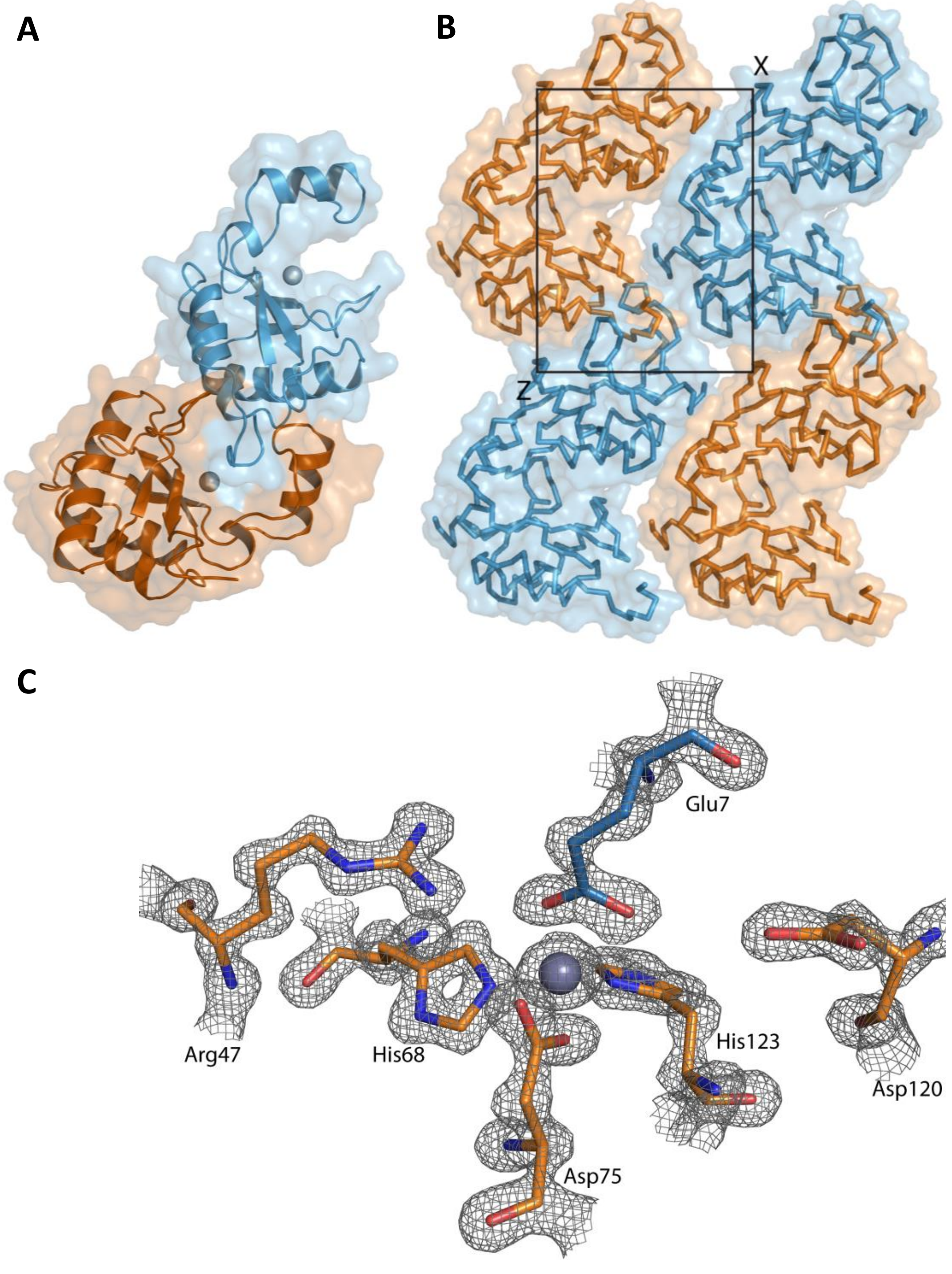

Supp. Figure S4: The asymmetric unit.

(A) The asymmetric unit contains two ChiX molecules, coloured in orange or blue and represented as cartoons with transparent surface. The molecules are linked between the open Zinc-binding cleft and the N-terminal loop region of the adjacent ChiX. **(B)** A view of the crystal packing showing four asymmetric units, with unit cell axes illustrated by black lines. ChiX molecules form filaments, with both free sides of the asymmetric unit interacting with the adjacent asymmetric unit through the same N-terminal loop to active site region. **(C)** Electron density around the Zinc ion.

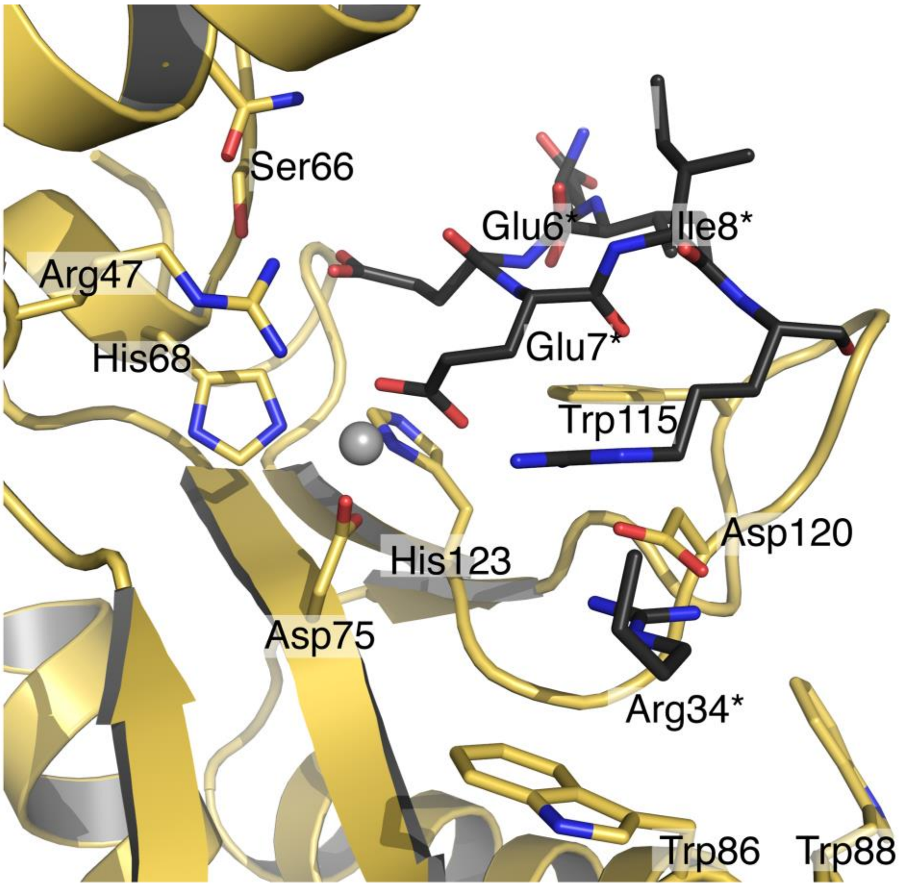

Supp. Figure S5: Predicting the substrate binding mechanism.
A cartoon and stick model highlighting residues that line, and are close to, the Zinc binding site cleft. Residues from the adjacent ChiX molecule in the asymmetric unit are coloured black.

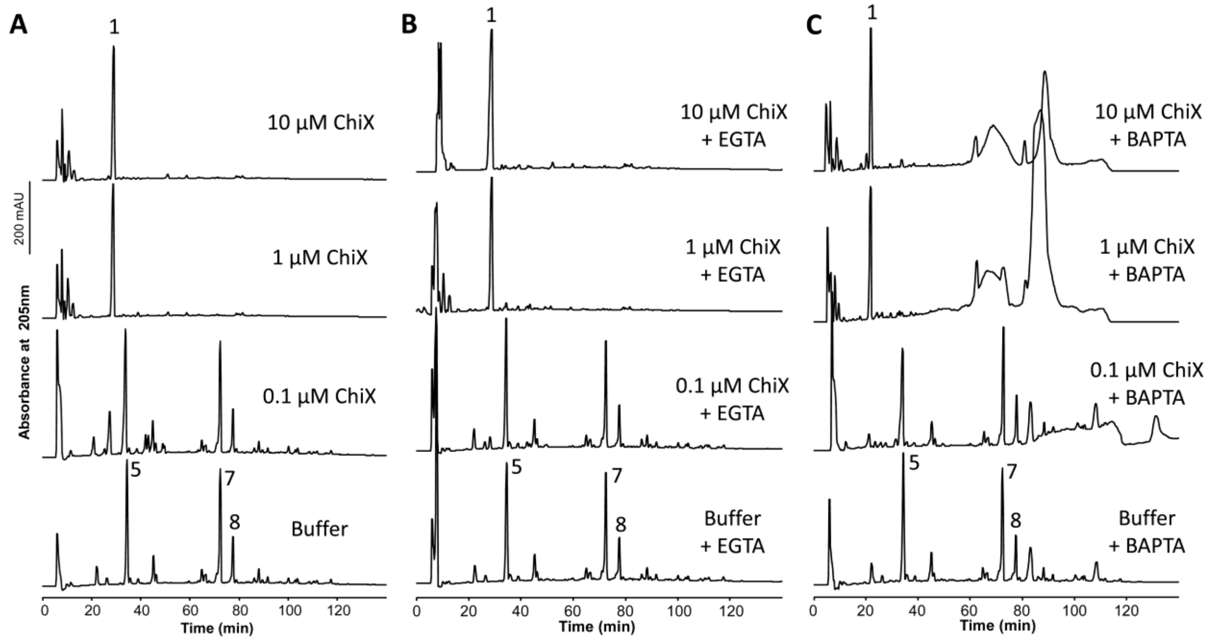

Supp. Figure S6. ChiX is not inhibited by EGTA or BAPTA.

Peptidoglycan from *E. coli* D456 was incubated with ChiX in the absence of chelator **(A)** or in the presence of EGTA **(B)** or BAPT **(C)** (both 20 mM), followed by digestion with cellosyl, reduction with sodium borohydride and separation of muropeptides by HPLC. The main product of ChiX (peak 1) and three of the major muropeptides present in the substrate (peaks 5, 7and 8) are penta, tetra-tetra and tetra-penta peptides. The presence of BAPTA in samples caused accumulation of broad peaks over the runs (in C), but EGTA or BAPTA did not inhibit ChiX.

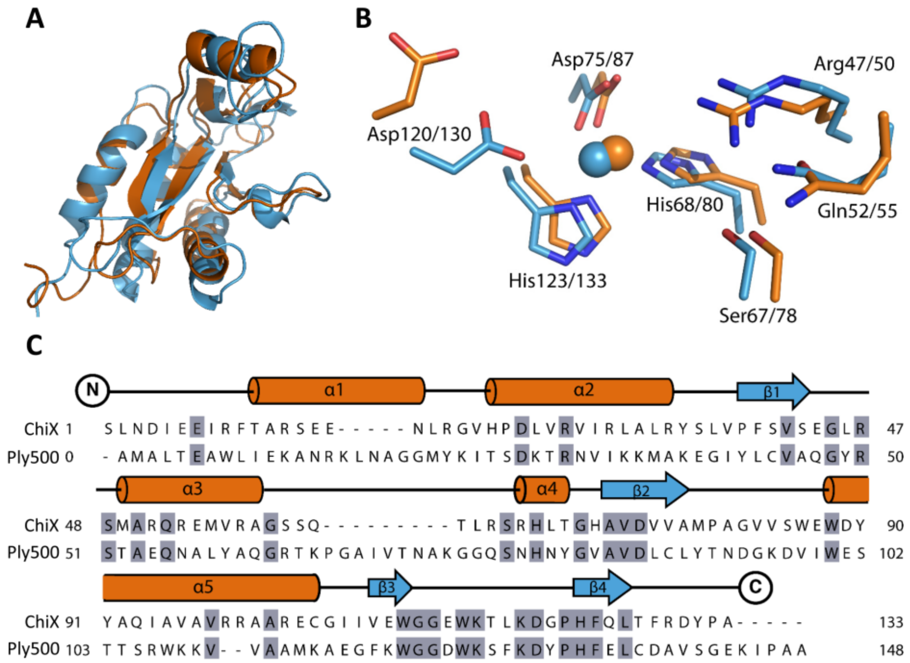

Supp. Figure S7: Active site similarities between ChiX and Ply500

Listeria phage protein Ply500 (blue), was identified as the most similar protein of known structure to *S. marcescens* ChiX (orange). **(A)** ChiX and Ply500 as cartoons showing conservation of secondary structure organization. ChiX exhibits a core structure (RMSD - 2.03 over 88 %) consistent with that of *Listeria* phage protein Ply500. **(B)** The active site region in stick form. **(C)** A sequence alignment with the ChiX sequence beginning '1' shown above the Ply500 sequence beginning '0'. The secondary structure elements for the ChiX protein are depicted above the sequence alignment.
